# Rupture of the Heart and Sudden Death

**Published:** 1844-01-27

**Authors:** Henry T. Taylor


### RUPTURE OF THE HEART AND SUDDEN DEATH.

BY HENRY T. TAYLOR, M. D.

I send you the particulars of a case of rupture of the heart, which, I think, will be worth communicating, as examples of it occur rarely, and may then become the subject of medico-legal inquiry, as in this instance:—Mrs. Keele, aged 53 years, the wife of a farmer's labourer, was taken ill suddenly on the morning of the 20th ult., and died in the course of half an hour. On my arrival the body was still warm, but afforded no indication of the cause of death, I therefore obtained an order from the coroner for a post-mortem examination. In answer to my inquiries the husband of the deceased stated that she had been in very good health, and only the day before had walked five miles with a burthen, but she had complained frequently of a bronchocele, which embarrassed her breathing at times, and to which he attributed her death. In December, 1842, she was ill with fever, and had an inflammation of the chest (but no decided information can be obtained on this point.)

At half-past 3, A. M., of the above mentioned day she was awakened by severe cough and difficulty of breathing. After making signs of great suffering about the throat, she "reared up" suddenly, and fell forward on the bed; when he obtained assistance she was quite dead. Two hours after death I found that the body had been laid out; it was that of a person of middle stature and spare conformation; the limbs were relaxed, and the face and general surface very pallid. There was a goîtrous tumour, which was not tense, nor of a size to justify the inference of death from that cause.

*Autopsy thirty hours after death.*—The body was now quite rigid and much congested in the depending parts; there was no œdema of the lower extremities. On the sternum being raised the lungs collapsed moderately; the pericardium was quite flaccid, and not at all encumbered with fat; the heart was found occupying its normal position, but obviously enlarged and much charged with fat. The right ventricle was collapsed, and on the middle of its anterior surface, close to the septum, I observed a depression, which proved to be a perforation into its cavity, large enough to admit a goose quill, and showing one of the fleshy columns through it. About an ounce and a half of fluid blood was effused into the pericardium; the inner surface of this membrane was marked by two small white patches of lymph anteriorly, but was otherwise healthy. The heart was removed, the usual quantity of dark blood flowing from the divided vessels: its weight was eleven ounces (avoirdupois.) There was a thick deposit of yellow fat under the serous covering, most abundant at the base and in the course of the coronary arteries; also on the anterior surface. The superficial vessels were much injected. On the cavities being laid open, the muscular tissue appeared somewhat pale and softened, its fibres being intermixed with fat. The right ventricle rather dilated, and its walls much thinned, so that near the point of rupture their thickness did not exceed half a line, and here the accumulation of fat was greatest. The opening was of irregular shape, with a torn edge and its greatest diameter transverse, and was situated two lines from the septum. The endocardium and valves were not at all diseased.

The left ventricle, which contained no coagula, was ten lines thick in the middle of its wall; the lining membrane of the aorta in its commencement was dyed of a bright red colour, and marked with numerous white spots. The aortic valves presented the same appearances, and there was several patches of redness on the musculi papillaris.

The lungs were everywhere adherent to the costal pleura inferiorly; the surface of a deep red, and near the anterior margins studded with emphysematous vesicles. The tissue of the lung was highly congested, but crepitated well on pressure.

The bronchi contained much mucus, and their mucous membrane was injected, as well as that of the trachea. The bronchial glands were enlarged, and many of them filled with calcareous matter.

The bronchocele consisted chiefly of enlargement of the central portion of the thyroid body, and was the size of an orange. A section of it exhibited the usual cellular structure, with a fibrinous centre. The other cavities were not examined.

The only comment that I have to make on the case is that a very small quantity of blood was effused from a considerable breach in the ventricle, whence I conclude that death must have been instantaneous from the heart's action being suspended by pressure of the sudden effusion.—*London Lancet, Nov.* 1843.

